# The Cytokine Profile in Acute Chikungunya Infection is Predictive of Chronic Arthritis 20 Months Post Infection

**DOI:** 10.3390/diseases6040095

**Published:** 2018-10-20

**Authors:** Aileen Y. Chang, Sarah Tritsch, St. Patrick Reid, Karen Martins, Liliana Encinales, Nelly Pacheco, Richard L. Amdur, Alexandra Porras-Ramirez, Alejandro Rico-Mendoza, Guangzhao Li, Jin Peng, Gary S. Firestein, Gary L. Simon, Jeff M. Bethony

**Affiliations:** 1 Department of Medicine, The George Washington University, Washington, DC 20037, USA; ramdur@mfa.gwu.edu (R.L.A.); gsimon@mfa.gwu.edu (G.L.S.); 2 Department of Microbiology, Immunology, and Tropical Medicine, The George Washington University, Washington, DC 20037, USA; Sarahtritsch@email.gwu.edu (S.T.); rachelli@email.gwu.edu (G.L.); pj_2011@gwu.edu (J.P.); jbethony@email.gwu.edu (J.M.B.); 3 Department of Pathology and Microbiology, University of Nebraska Medical Center, Omaha, NE 68198, USA; Patrick.reid@unmc.edu; 4 Army Medical Research Institute for Infectious Diseases, Fort Detrick, MD 21702, USA; Karen.a.martins2.ctr@mail.mil; 5 Department of Medicine, Allied Research Society LLC, Barranquilla, 080020, Atlántico, Colombia; l.encinalesmd@alliedresearchsociety.com (L.E.); nepamer20@hotmail.com (N.P.); 6 Grupo de Medicina Comunitaria y Salud Colectiva, Universidad El Bosque, 110121, Bogotá, Colombia; porras.alexandra@gmail.com (A.P.-R.); aricomendoza@gmail.com (A.R.-M.); 7 Department of Medicine, University of California, San Diego, La Jolla, CA 92093, USA; Gfirestein@ucsd.edu

**Keywords:** arthritis, chikungunya, cytokine, alphavirus

## Abstract

The cytokine profile during acute chikungunya infection that predicts future chronic arthritis has not yet been investigated. We conducted a nested case-control study comparing serum cytokine concentrations during acute chikungunya infection in cases (*n* = 121) that reported the presence of chronic joint pain versus age- and gender-matched controls (*n* = 121) who reported recovery at 20 months post infection. We observed that a robust cytokine response during acute infection was correlated with a decreased incidence of chronic joint pain and that low TNFα, IL-13, IL-2, and IL-4 during acute infection was predictive of chronic joint pain. These data suggest that a robust cytokine response is necessary for viral clearance and cytokines that are related to immune tolerance during acute infection may be protective for chronic arthritis pathogenesis.

## 1. Introduction

Chikungunya virus (CHIKV) is an alphavirus that is spread by mosquitos, infects an estimated 1 million people annually [1], and causes a chronic debilitating arthritis in one fourth of infected individuals [2]. CHIKV-induced arthritis is associated with a high initial viral load and prolonged viral infection [3], suggesting that the joint symptoms may result from either failure of the innate immune system to clear the virus or post-viral, immune-mediated inflammatory responses. Since the mechanism of CHIKV chronic arthritis pathogenesis is not well-established, there is no standard treatment for this debilitating condition.

To better understand the immune factors during acute CHIKV infection that contribute to chronic arthritis pathogenesis, we conducted a nested case-control study comparing serum cytokine concentrations during acute chikungunya infection in cases (*n* = 121) that reported the presence of chronic joint pain versus age- and gender-matched controls (*n* = 121) who reported recovery at 20 months post infection. Our hypothesis was that CHIKV cases with higher levels of cytokines that mediate acute inflammation (IL-6, IL-1β, TNF-α) or stimulate humoral (IL-5) or cellular (IL-12) inflammation during acute infection would be more likely develop chronic joint pain. Interestingly, we observed that a robust cytokine response during acute infection was correlated with a decreased incidence of chronic joint pain and, specifically, that low TNFα, IL-13, IL-2, and IL-4 during acute infection was predictive of chronic joint pain.

## 2. Materials and Methods

### 2.1. Study Design

As previously reported [2], 500 patients with clinically confirmed chikungunya virus infection were enrolled as part of a prospective cohort in January 2015. Briefly, a diagnosis of chikungunya virus was serologically confirmed via IgM and IgG antibody capture enzyme-linked immunosorbent assay (ELISA). A baseline 33-item survey was conducted to ascertain demographic characteristics, exposure history, and symptoms. A subsequent 56-item telephone survey was performed a median of 20 months after infection and included an assessment of the character and duration of persistent chikungunya arthritis symptoms.

For this nested case-control analysis, we matched each case that reported chronic arthritis that was attributed to their CHIKV infection at a median of 20 months post infection with an age- and gender-matched CHIKV control patient without chronic arthritis at the same timepoint. Serum cytokine concentrations during acute infection were compared in cases versus controls.

### 2.2. Ethics Statement

All subjects gave their informed consent for inclusion before they participated in the study. The study was conducted in accordance with the Declaration of Helsinki, and the protocol was approved by the Ethics Committee of Universidad El Bosque (Protocol: Vigilancia centinela de eventos infecciosos en Colombia).

### 2.3. Setting

The patients were referred to the study from the Sabanalarga, Barranquilla, Juan de Acosta, Manatí, Luruaco, and Baranoa municipalities in the Atlántico Department, Colombia.

### 2.4. Participants

Patients with clinical symptoms of chikungunya with a fever of >38 °C, severe joint pain or arthritis, and acute onset of erythema multiforme, with symptoms not explained by other medical conditions, and serologic confirmation of CHIKV infection were included.

### 2.5. Serum Cytokine Evaluation

Sera were stored at −80 °C until analysis. Evaluation of the serum cytokine concentration of interleukin (IL)-10, IL-1β, IL-6, tumor necrosis factor (TNF)-α, IL-12 (p70), IL-13, IL-17, IL-2, IL-4, and IL-5 was performed using the Milliplex^R^ MAP Kit High Sensitivity Magnetic Bead Panel on a MAGPIX^R^ powered by Luminex^R^ XMAP technology.

### 2.6. Statistical Analysis

Cytokine distributions were examined with histograms and all were highly positively skewed, often with outliers. *p*-values for the Shapiro–Wilks test of non-normality, QQ plots, and the mean and standard deviation versus median and interquartile range were examined. The QQ plot for normally distributed variables shows that the data points were aligned in a linear arrangement (age plot), while the shape becomes either S-shaped or J-shaped when there is non-normality and/or outliers as shown in the graphs. The Kruskal–Wallis test was used to test for differences in the distribution location (median) for patients with versus without joint pain, for each cytokine. Each cytokine was significantly non-normal (all *p* < 0.0001). We therefore coded each cytokine level into quintiles. The association of a cytokine quintile with joint pain was examined with a chi-square test and graphed. Spearman correlations between cytokine levels were examined. To understand which cytokines had independent associations with joint pain, we used a multivariable logistic regression model, also including age and sex as covariates. Stepwise selection was used with *p* < 0.20 required for model entry and exit. Based on the final model, we calculated each subject’s probability of having joint pain using the logistic model. The probability values were coded into quintiles, and the association of the probability quintiles with joint pain was examined with a chi-square test. Statistical analyses were conducted using SAS software, version 9.4 (SAS Institute Inc., Cary, NC, USA).

## 3. Results

**Figure 1:**
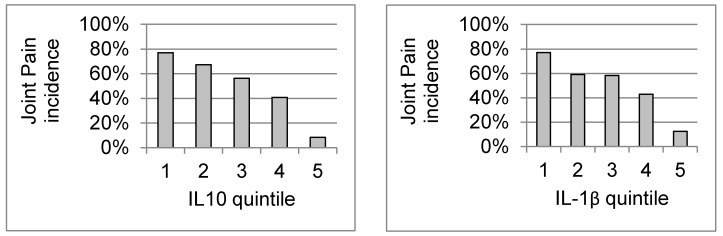

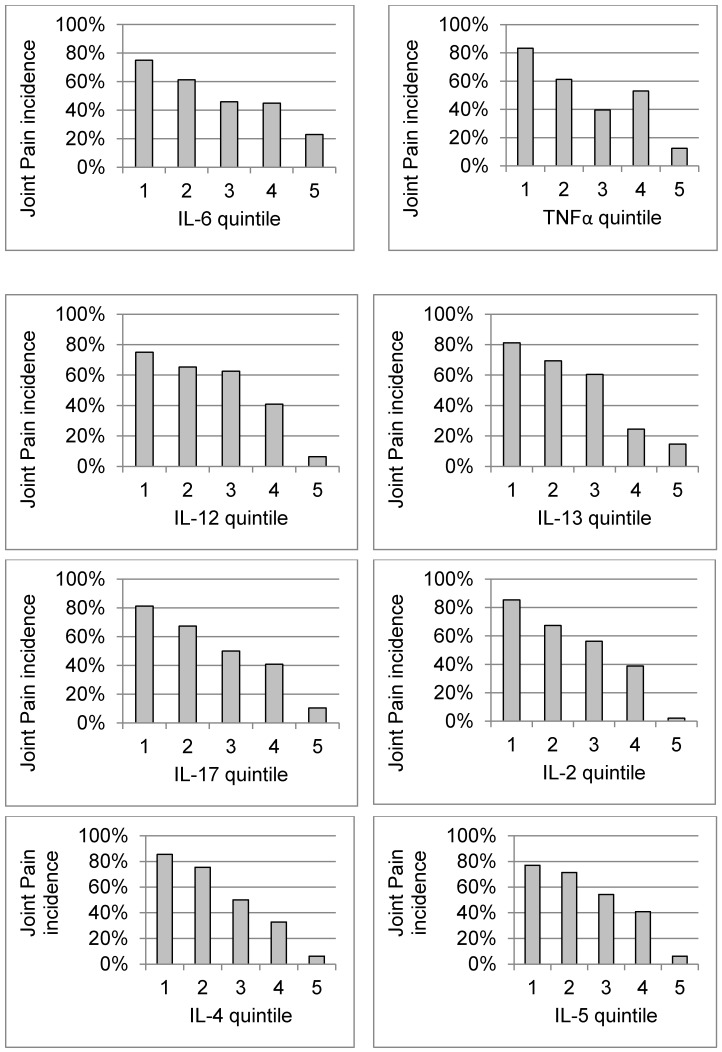
Association of cytokine quintiles with joint pain.

The serum cytokine levels (Supplemental Table S1) of age- and gender-matched patients with (*n* = 121) and without (*n* = 121) chronic arthritis 20 months post infection were compared (Table 1 Patient Characteristics). All of the cytokines were detected in all of the patients. Distribution statistics are shown in Supplemental Table 1, and the frequency histograms and QQ plots for each cytokine are shown in Supplemental Figure 1. Statistics and graphs for age are also shown as a comparison, since this variable was normally distributed. In general, mean levels were 2 to 4 times higher in those patients without joint pain, and median levels were generally 50% to 100% higher (all *p* < 0.0001) (Supplemental Table S2). In each graph, with increasing cytokine quintile, the number of patients who are positive for joint pain decreases, while the number who are negative for joint pain increases (Supplemental Table S2). Each cytokine was significantly negatively associated with joint pain (all *p* < 0.0001) (Figure 1). Spearman r’s in general were in the range of 0.40 to 0.75 and all were significant (all *p* < 0.0001). None of the Spearman r’s between any cytokines and age or sex were significant. Spearman r’s between cytokines and joint pain were all significant (all *p* < 0.0001) and ranged from −0.57 (for IL-4) and −0.55 (for IL-2) to −0.41 (for IL-1β) and −0.34 (for IL-6).

**Figure 2:**
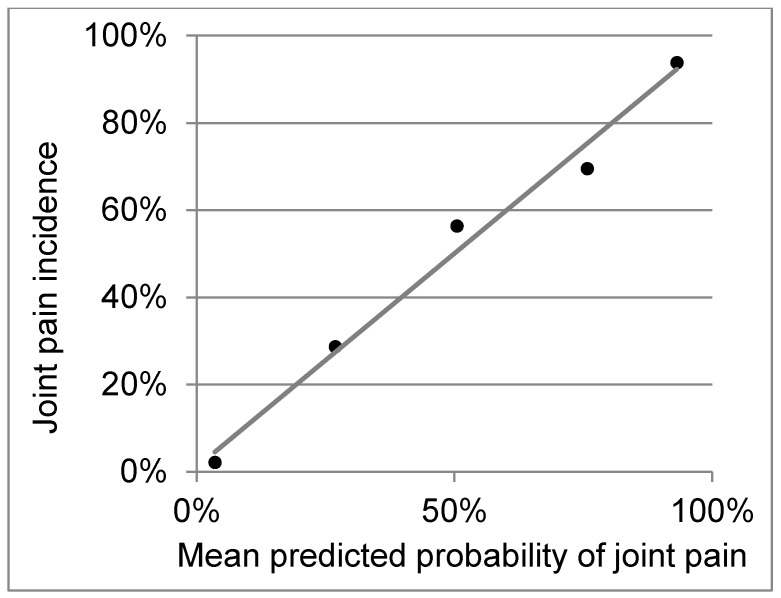
Calibration of the logistic regression model’s risk scores versus incidence of joint pain. There is very strong correspondence between the predicted probability and the observed incidence of joint pain across risk quintiles.

The logistic regression model had an area under the curve (AUC) of 0.88, and the final model included TNFα, IL-13, IL-2, and IL-4 (Table 2). Calibration was excellent, with very strong correspondence between the predicted probability and the observed incidence of joint pain, across risk quintiles (Figure 2). In the examination of cytokine patterns, for the purpose of allowing for the best differentiation of cytokine patterns, we do not include the other cytokines, which are more weakly associated with joint pain. What we found is that the patterns themselves are similar between patients with versus without joint pain; however, those with joint pain simply have lower baseline levels across all of the cytokines (Supplemental Figure S3).

## 4. Discussion

This is the largest age- and gender-matched analysis to report that the cytokine profile is predictive of chronic arthritis after CHIKV infection. We observed that a robust cytokine response during acute infection was correlated with a decreased incidence of chronic joint pain and that lower TNFα, IL-2, IL-4, and IL-13 during acute infection was predictive of chronic joint pain. These data suggest that a robust cytokine response is necessary for viral clearance; in particular, TNFα and cytokines that are related to immune tolerance during acute infection may be protective for chronic arthritis pathogenesis.

The available studies have reported similar results, suggesting that chronic arthritis is associated with deregulation of inflammation during acute and convalescent phases, with decreases in Th2 cytokines and increases in Th1 cytokines in those with persistent symptoms [3,4,5,6]. Our findings are also consistent with findings from Hoarau et al., who found that the Th2 cytokines IL-4 and IL-13 tended to be produced in smaller amounts during the acute phase in patients progressing to chronic disease; however, their comparisons were not statistically significant, perhaps due to the small sample size of 15 people [3].

We also found that IL-2 was decreased during acute infection in patients progressing to chronic disease. This may be of importance, as IL-2 is known to prevent autoimmune disease by promoting the differentiation of naïve T cells into regulatory T cells [7]. CHIKV arthritis is characterized by an infiltration of large numbers of CD4^+^ T cells into the joint tissue [3], and CD4^+^ T cells are known to be important in CHIKV joint swelling [8] and chronic joint pain (3). Failure of cell-mediated immune tolerance may contribute to CHIKV arthritis pathogenesis. Recombinant IL-2, aldesleukin, has been approved for the treatment of cancers [9] and has been successful in the immune modulation of autoimmune diseases, such as type 1 diabetes and vasculitis [10]. Further research is needed to determine the implications for cytokine treatments in CHIKV arthritis.

One limitation of this analysis is that, given the case-control design with fifty percent prevalence of joint pain, this mathematical model may not necessarily be clinically useful to predict the incidence of chronic joint pain in populations with a lower prevalence of joint pain. The primary value of this analysis is to provide insights into the pathophysiologic mechanisms of chronic CHIKV arthritis pathogenesis.

## Figures and Tables

**Table 1:** Patient characteristics 20 months post chikungunya infection.

Patient Characteristic	With Joint Pain (*n* = 121)	Without Joint Pain (*n* = 121)
Age, mean ± (SD) in years	49 ± 17	48 ± 17
Female, percent	89%	89%

**Table 2:** The final logistic regression model for cytokines.

Cytokine Quintile	Adjusted OR	95% Wald Confidence Limits		*p*	Parameter Estimate (se)
TNFα	0.650	0.487	0.866	0.0033	−0.43 (0.15)
IL-13	0.799	0.567	1.125	0.1984	−0.22 (0.17)
IL-2	0.573	0.401	0.819	0.0023	−0.56 (0.18)
IL-4	0.504	0.372	0.683	<0.0001	−0.68 (0.15)
Intercept				<0.0001	3.66

Associations with joint pain. The equation to calculate the probability of having joint pain was risk = 3.66, –0.43(TNFα), –0.22(IL-13), –0.56(IL-2), –0.68(IL-4), where the quintile (scored 0–4) is used for each cytokine. Probability = exp(risk)/(1 + exp(risk)). OR, odds ratio.

## Supplementary Materials

The supplementary materials are available online http://www.mdpi.com/2079-9721/6/4/95/s1.
