# Supplementary material for: The Cytokine Profile in Acute Chikungunya Infection is Predictive of Chronic Arthritis 20 Months Post Infection
Source: Diseases. 2018 Oct 20;6(4):95. doi: 10.3390/diseases6040095 (PMC6313749; doi:10.3390/diseases6040095)

diseases

MDPI

Article

Cytokine profile in acute chikungunya infection is predictive of chronic arthritis 20-months post-infection

Aileen Y. Chang [1,]*, Sarah Tritsch [2], St. Patrick Reid [3], Karen Martins [4], Liliana Encinales [5], Nelly Pacheco [5], Richard L. Amdur [1], Alexandra Porras-Ramirez [6], Alejandro Rico-Mendoza [6], Guangzhao Li [2], Jin Peng [2], Gary S. Firestein [7], Gary L. Simon [1] and Jeff M. Bethony [2]

Supplemental Tables

Supplemental Table 1. Normality tests for cytokines and age. Cytokines all have significant positive skew, while age is normally distributed.

Cytokine	Mean ± sd	Median [IQR]	Skewness	Non-normality (Shapiro-Wilk test p)
IL-10	20.6 ± 28.3	9.3 [4.7-15.4]	1.9	<.0001
IL-1β	8.9 ± 18.6	3.4 [2.0-6.0]	5.7	<.0001
IL-6	7.1 ± 12.0	2.7 [1.6-8.7]	4.8	<.0001
TNF-α	12.6 ± 11.4	7.8 [5.2-14.7]	1.8	<.0001
IL-12	11.6 ± 12.3	6.9 [4.2-10.1]	2.1	<.0001
IL-13	7.7 ± 9.7	5.1 [2.6-8.8]	4.9	<.0001
IL-17	16.2 ± 16.3	10.7 [5.4-17.7]	1.9	<.0001
IL-2	6.6 ± 9.0	3.1 [1.7-5.0]	2.0	<.0001
IL-4	51.9 ± 45.2	34.8 [22.9-59.9]	1.4	<.0001
IL-5	7.8 ± 10.1	4.1 [2.6-6.0]	2.0	<.0001
AGE	48.6 ± 16.9	48.0 [37.0-60.0]	0.1	0.21

Supplemental Table 2. Distribution of cytokines, stratified by joint pain. Kruskal-Wallis test shows significant non-overlap for all cytokine distributions for those patients with vs without joint pain.

	No joint pain		Had joint pain		Kruskal-Wallis Test
**Cytokine**	**Mean ± sd**	**Median [IQR]**	**Mean ± sd**	**Median [IQR]**	***p*-value**
IL-10	33.0 ± 35.0	13.6 [8.6-66.5]	8.1 ± 8.2	6.7 [3.5-10.1]	<.0001
IL-1β	13.9 ± 24.2	4.5 [2.8-18.1]	3.9 ± 7.9	2.6 [1.7-3.9]	<.0001
IL-6	9.0 ± 12.2	4.9 [2.2-11.4]	5.1 ± 11.6	2.0 [1.2-3.8]	<.0001
TNF-α	17.0 ± 13.0	9.9 [6.9-28.7]	8.3 ± 7.5	6.0 [4.2-9.9]	<.0001
IL-12	17.4 ± 16.4	8.9 [5.8-31.7]	5.8 ± 4.0	5.3 [3.4-7.4]	<.0001
IL-13	10.4 ± 8.3	8.0 [4.9-16.2]	5.0 ± 10.2	3.5 [1.9-5.3]	<.0001

IL-17	23.5 ± 19.4	14.2 [10.6-38.3]	8.8 ± 6.9	7.6 [4.2-11.4]	<.0001
IL-2	10.9 ± 11.1	4.6 [3.0-21.2]	2.3 ± 1.7	2.0 [1.2-3.2]	<.0001
IL-4	75.5 ± 51.2	50.5 [33.9-133.5]	28.4 ± 19.0	25.0 [17.3-35.8]	<.0001
IL-5	12.4 ± 12.6	5.5 [3.8-23.9]	3.2 ± 2.0	3.1 [1.8-4.3]	<.0001

Supplemental Figures

Supplemental Figure 1. Frequency histograms and QQ plots for cytokines and age.

Cytokine	Histogram	QQ-plot
IL-10	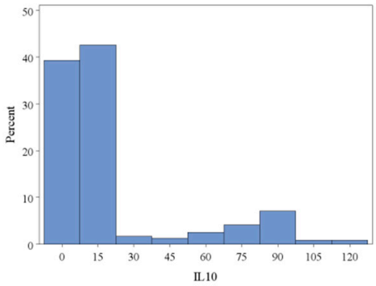	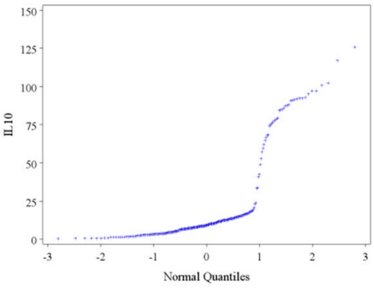
IL-1β	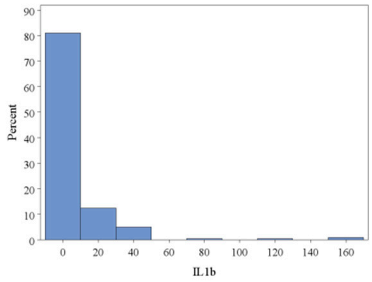	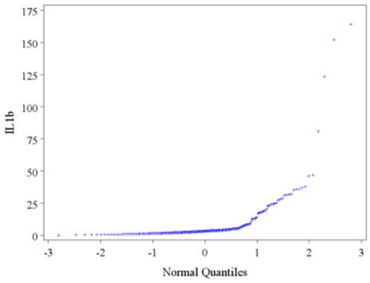
IL-6	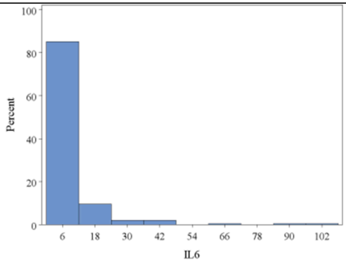	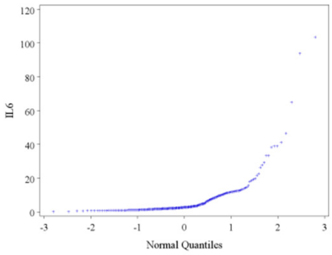
TNF-α	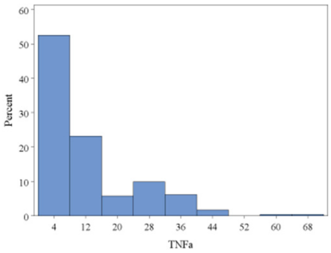	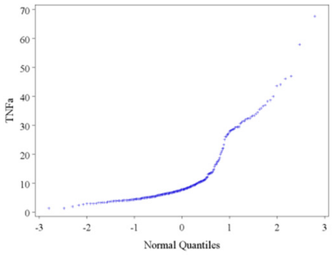
IL-12	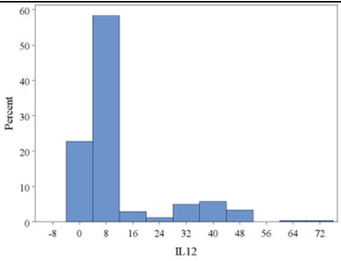	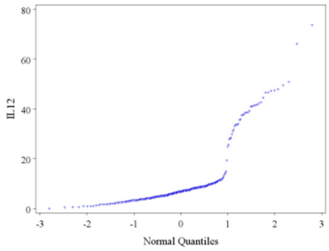

IL-13

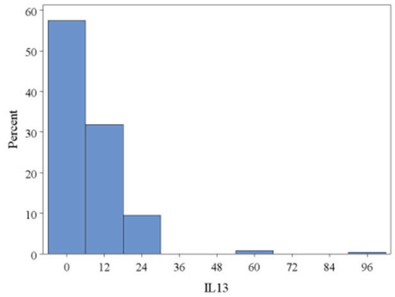

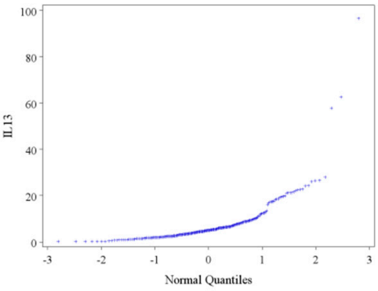

IL-17

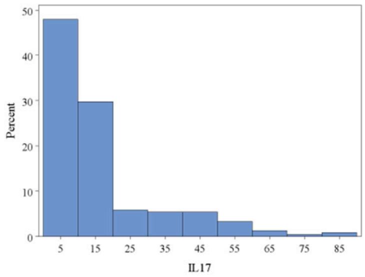

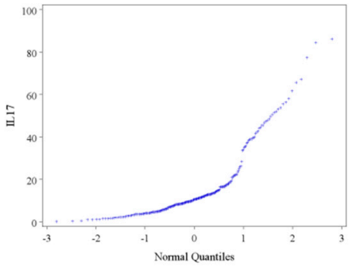

IL-2

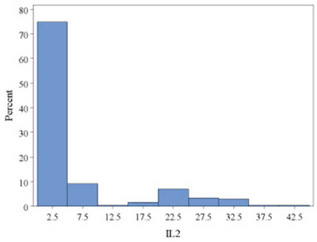

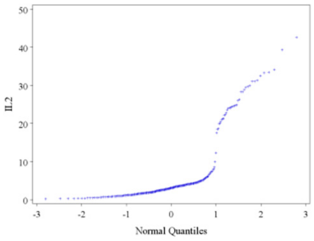

IL-4

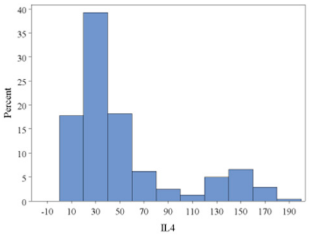

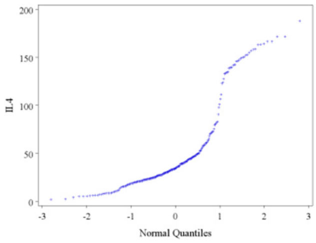

IL-5

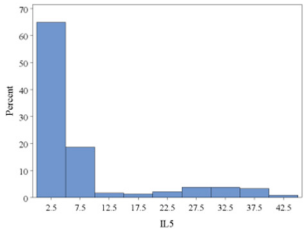

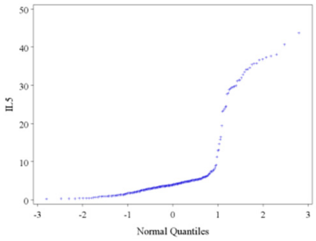

AGE

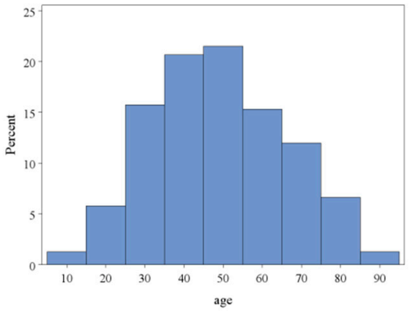

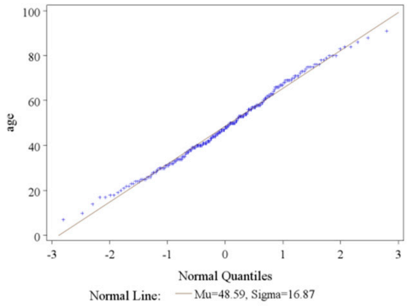

Supplemental Figure 2. Distribution of cytokine quintiles by joint pain.

<table>
<tr><th>Cytokine</th><th>Distribution by joint pain status</th></tr>
<tr><td>IL-10</td><td>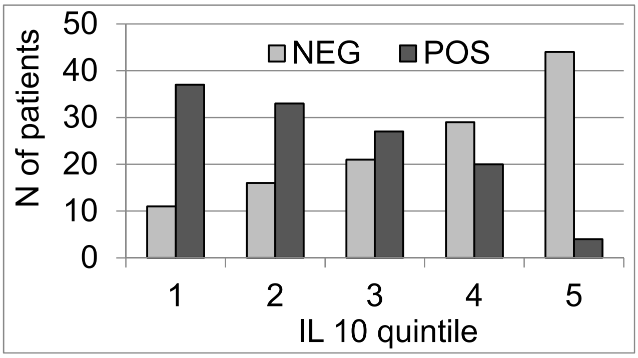
</td></tr>
<tr><td>IL-1β</td><td>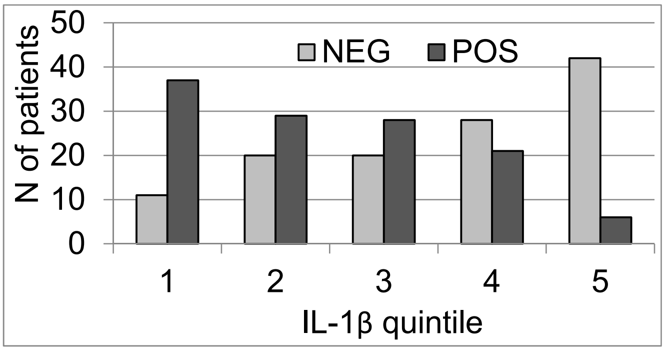
</td></tr>
<tr><td>IL-6</td><td>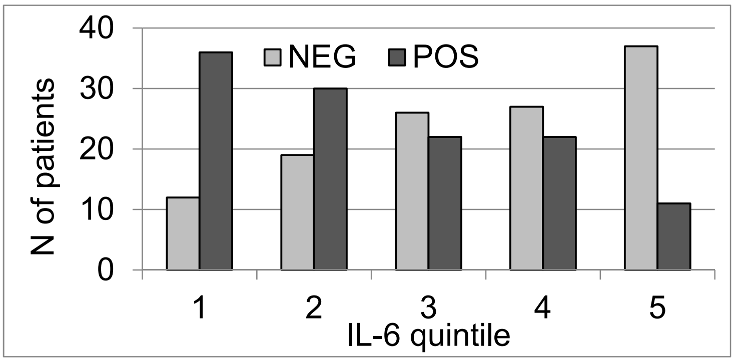
</td></tr>
<tr><td>TNF-α</td><td>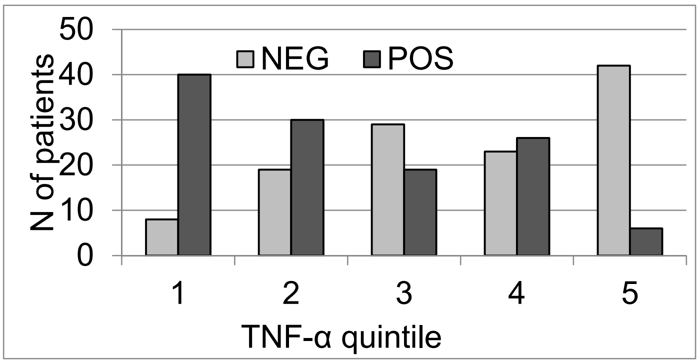
</td></tr>
</table>

IL-12	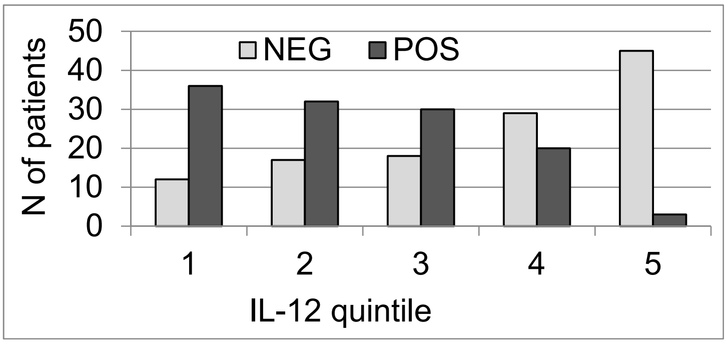
IL-13	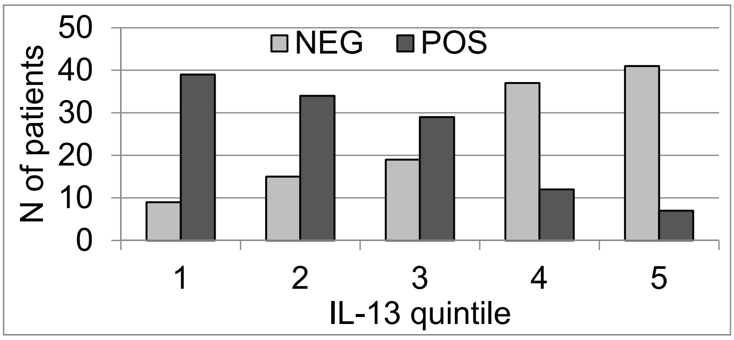
IL-17	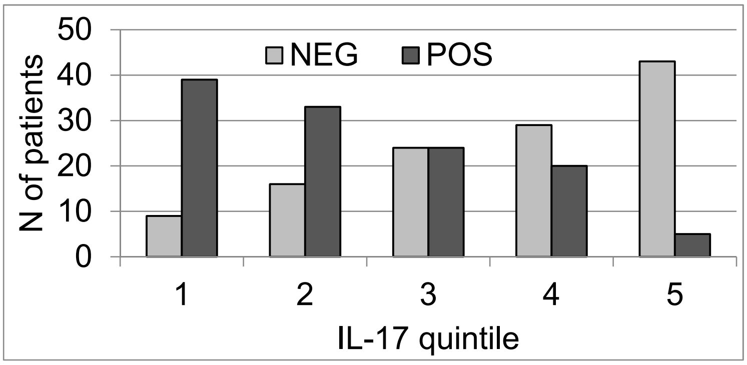
IL-2	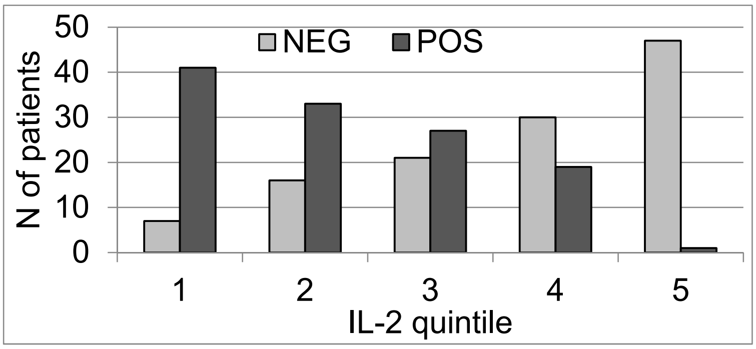

<table>
<tr><td>IL-4</td><td>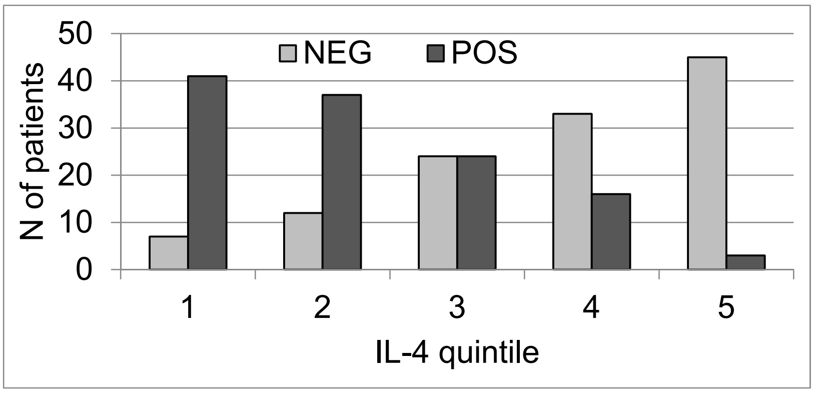
</td></tr>
<tr><td>IL-5</td><td>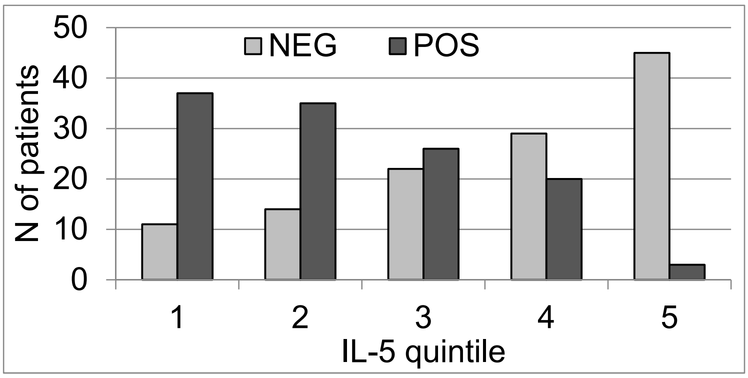
</td></tr>
</table>

Supplemental Figure 3. Pattern of mean baseline quintile for four cytokines, stratified by later joint pain. The cytokine profiles are similar in shape, but patients with joint pain have lower baseline levels for each cytokine. Error bars show the 95% confidence interval for mean quintile.

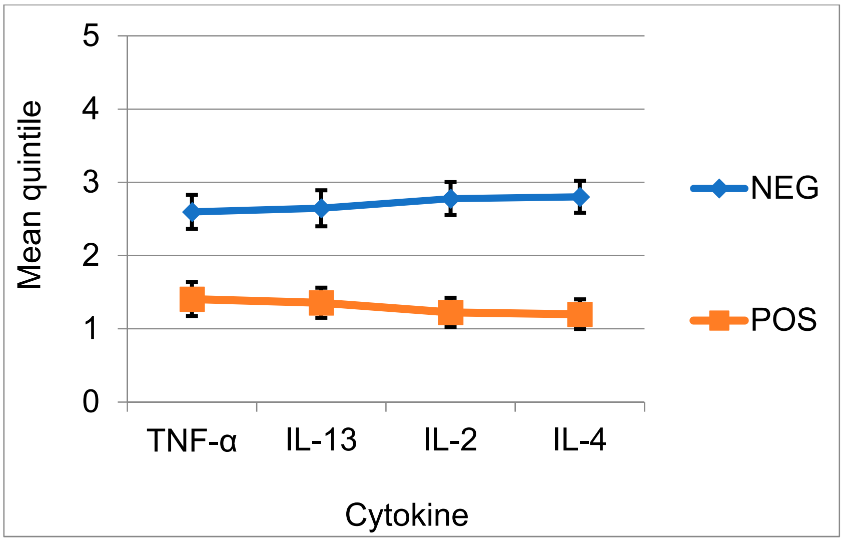

Supplement: Supplementary file 1 [file diseases-06-00095-s001.pdf]
